# High penetrance of acute intermittent porphyria in a Spanish founder mutation population and *CYP2D6* genotype as a susceptibility factor

**DOI:** 10.1186/s13023-019-1031-7

**Published:** 2019-02-26

**Authors:** María Barreda-Sánchez, Juan Buendía-Martínez, Guillermo Glover-López, Carmen Carazo-Díaz, María Juliana Ballesta-Martínez, Vanesa López-González, María José Sánchez-Soler, Lidya Rodriguez-Peña, Ana Teresa Serrano-Antón, Remedios Gil-Ferrer, Maria del Carmen Martínez-Romero, Pablo Carbonell-Meseguer, Encarna Guillén-Navarro

**Affiliations:** 1 Cátedra de Genética (Pabellón 9), Facultad de Ciencias de la Salud, Universidad Católica de Murcia (UCAM), Avda. Los Jerónimos s/n, CP 30107 Guadalupe, Murcia, Spain; 2 Servicio de Neurología, Hospital Comarcal del Noroeste, Caravaca, Murcia, Spain; 3 Centro de Bioquímica y Genética Clínica, Hospital Clínico Universitario Virgen de la Arrixaca, IMIB- Arrixaca, Murcia, Spain; 4 CIBERER-ISCIII, Madrid, Spain; 5 Grupo Applied Statistical Methods in Medical Research, Universidad Católica de Murcia (UCAM), Murcia, Spain; 6 Sección Genética Médica (Pabellón Materno-Infantil), Servicio de Pediatría, Hospital Clínico Universitario Virgen de la Arrixaca, IMIB-Arrixaca, Ctra. Madrid-Cartagena s/n, CP 30120 El Palmar, Murcia, Spain; 7 Sección Genética Médica, Servicio de Pediatría, Hospital Clínico Universitario Virgen de la Arrixaca, Universidad de Murcia, IMIB-Arrixaca, Murcia, Spain; 8 Sección Genética Médica, Servicio de Pediatría, Hospital Clínico Universitario Virgen de la Arrixaca, Murcia, Spain; 9 Centro de Bioquímica y Genética Clínica, Hospital Clínico Universitario Virgen de la Arrixaca, IMIB-Arrixaca, Murcia, Spain; 10 Departamento de Cirugía, Pediatría, Obstetricia y Ginecología, Facultad de Medicina, Universidad de Murcia, Murcia, Spain

**Keywords:** Acute intermittent porphyria, Founder mutation, Penetrance, *CYP2D6*, Susceptibility factor, Acute attacks, Personalized medicine, Genomic medicine

## Abstract

**Background:**

Acute intermittent porphyria (AIP) is a low-penetrant genetic metabolic disease caused by a deficiency of hydroxymethylbilane synthase (HMBS) in the haem biosynthesis. Manifest AIP (MAIP) is considered when carriers develop typical acute neurovisceral attacks with elevation of porphyrin precursors, while the absence of attacks is referred to as latent AIP (LAIP). Attacks are often triggered by drugs, endocrine factors, fasting or stress.

Although AIP penetrance is traditionally considered to be around 10–20%, it has been estimated to be below 1% in general population studies and a higher figure has been found in specific AIP populations. Genetic susceptibility factors underlying penetrance are still unknown.

Drug-metabolizing cytochrome P450 enzymes (CYP) are polymorphic haem-dependent proteins which play a role in haem demand, so they might modulate the occurrence of AIP attacks.

Our aim was to determine the prevalence and penetrance of AIP in our population and analyse the main hepatic *CYP* genes to assess their association with acute attacks. For this, CYP2C9*2, *3; CYP2C19*2; CYP2D6*4, *5; CYP3A4*1B and CYP3A5*3 defective alleles were genotyped in fifty AIP carriers from the Region of Murcia, a Spanish population with a high frequency of the *HMBS* founder mutation c.669_698del30.

**Results:**

AIP penetrance was 52%, and prevalence was estimated as 17.7 cases/million inhabitants. The frequency of defective *CYP2D6* alleles was 3.5 times higher in LAIP than in MAIP. MAIP was less frequent among CYP2D6*4 and *5 carriers (*p* < 0.05). The urine porphobilinogen (PBG)-to-creatinine ratio was lower in these individuals, although it was associated with a lower prevalence of attacks (p < 0.05) rather than with the *CYP2D6* genotype.

**Conclusions:**

AIP prevalence in our region is almost 3 times higher than that estimated for the rest of Spain. The penetrance was high, and similar to other founder mutation AIP populations. This is very relevant for genetic counselling and effective health care. CYP2D6*4 and *5 alleles may be protective factors for acute attacks, and *CYP2D6* may constitute a penetrance-modifying gene. Further studies are needed to confirm these findings, which would allow a further progress in clinical risk profile assessment based on the *CYP* genotype, leading to predictive personalized medicine for each AIP carrier in the future.

## Background

Acute intermittent porphyria (AIP, MIM # 176000) is an autosomal dominant disorder caused by a deficiency of hydroxymethylbilane synthase (HMBS; EC 2.5.1.61), which catalyzes the third step of haem biosynthesis. AIP is the most common acute porphyria, with a prevalence of 5.9 cases/million inhabitants in Europe as a whole and 6.3 cases/million inhabitants in Spain [1].

The main clinical manifestations are episodic acute neurovisceral attacks characterized by abdominal pain, vomiting, tachycardia, hypertension and dark urine.

AIP is a low-penetrant disease, and generally 10–20% of AIP carriers develop acute attacks [2–4], known as manifest AIP (MAIP), as opposed to latent AIP carriers (LAIP) with no attacks. However, studies based on the frequency of pathogenic variants in *HMBS* gene in the general population suggest significantly lower penetrance (< 1%) [5, 6]. On the other hand, higher penetrance has been associated with specific mutations, such as R173W and W198X. [7].

Acute attacks are more frequent in women and rarely occur before puberty [8, 9]. Such acute attacks are often triggered by precipitating factors, which include several drugs, alcohol, steroid hormones, infection or fasting. Genetic background might be involved in variations in penetrance, although the underlying genes involved are still unknown [10].

In the liver, free haem exerts negative feedback regulation of ubiquitous 5-aminolevulinic acid synthase (ALAS1; EC 2.3.1.37), the rate-limiting enzyme in haem biosynthesis [11–13]. Acute attacks occur when hepatic haem synthesis is overstimulated. This causes overproduction of the porphyrin precursors 5-aminolevulinic acid (ALA) and porphobilinogen (PBG), which have been associated with clinical manifestation of AIP, and increases in urinary excretion. After acute attack, ALA and PBG gradually decrease, although levels may remain high for time ranging from weeks to years [14, 15].

Drug-metabolizing cytochrome P450 enzymes (CYP) constitute the main hepatic haemoproteins [16]. CYP and ALAS1 synthesis is coordinated, while several drugs are transcriptional inducers of both genes [17, 18]. Since the turnover of CYP is a determining factor in the hepatic induction of haem synthesis, it might influence the penetrance of hepatic porphyrias.

Inter-individual differences in drug metabolism are common, in part due to inherited polymorphisms in *CYP* genes [19]. A high frequency of two polymorphisms in *CYP1A2* and *CYP1A1* genes has been reported in porphyria cutanea tarda, both polymorphisms being associated with increased enzymatic activity [20, 21]. In addition, non-functional CYP2D6*3 and *4 alleles seemed to be less frequent in AIP carriers compared to a control population [22].

We hypothesized that *CYP* genes might act as modifiers in AIP, and that specific alleles would constitute susceptibility factors for developing acute attacks. Taking advantage of our genetically highly homogeneous population (most AIP cases carry the founder mutation c.669_698del30 in the *HMBS* gene) [23], we aimed to identify AIP penetrance modifying *CYP* genes that act as risk factors for developing acute attack. The goal of this study was to determine AIP prevalence and penetrance in our region associated with the *HMBS* founder mutation and the frequency of common defective alleles that lead to a disturbance in main hepatic CYP enzymes [24], as well as to analyse their relationship with the occurrence of acute attacks in AIP. With this aim, CYP2C9*2, *3; CYP2C19*2; CYP2D *4, *5; CYP3A4*1B and CYP3A5*3 alleles were studied in a group of AIP carriers with a high prevalence of the aforementioned founder mutation.

## Methods

### Patients and clinical assessment

Fifty Spanish AIP genetic carriers, from 21 different families in the Region of Murcia (Southern Spain), were included in the study. Informed consent approved by the Research Ethics Board of the Hospital Clínico Universitario Virgen de la Arrixaca was obtained from all patients. Most of them (78%) carried the founder pathogenic variant NC_000011.9 (NM_000190.3):c.669_698del30 in *HMBS* gene responsible for AIP.

To determine the occurrence of acute attacks (MAIP frequency), patients were interviewed and clinical records were revised based on a systematic follow-up of AIP carriers currently carried out by the Medical Genetics Section of our hospital, which is the referral unit in our province. Patients who had history of at least one acute attack with a typical porphyrin precursor excretion profile, requiring hospitalization and treatment with haemin were classified as MAIP.

### CYP genotyping

DNA was isolated from blood samples with the QIAamp DNA Blood Mini kit (Qiagen, Hilden, Germany). Genotyping of CYP2C9*2 (rs1799853; NC_000010.10:g.96702047C > T), CYP2C9*3 (rs1057910; NC_000010.10:g.96741053A > C); CYP2C19*2 (rs4244285; NC_000010.10:g.96541616G > A); CYP2D6*4 (rs3892097; NC_000022.10:g.42524947C > T), CYP3A4*1B (rs2740574; NC_000007.13:g.99382096C > T) and CYP3A5*3 (rs776746; NC_000007.13:g.99270539C > T) alleles was performed with TaqMan® Drug Metabolism Genotyping Assays (Applied Biosystems, Foster City, USA). CYP2D6*5 allele, entailing *CYP2D6* gene deletion, was analysed with TaqMan® Copy Number Assay (Applied Biosystems). All assays were performed with available commercial pre-designed kits. Assays were run on an ABI® 7500 Fast Real-Time PCR System (Applied Biosystems) and analysed with 7500 Software and CopyCaller (Applied Biosystems).

Individuals carrying none of the analysed defective alleles were predicted to carry normal functional allele since other defective alternative alleles are rare in our population, such as CYP2C19*3 which may be frequent in Asian populations as opposed to Caucasian.

### Biochemical analyses

First morning urine samples were collected at a symptoms-free stage, at least after 6 months of an acute attack. Samples were available only in 45 patients. Urinary creatinine was analysed with a commercial kit (CREJ2) based on the Jaffé method in a Roche Cobas c system (Roche, Mannheim, Germany). ALA and PBG were measured by spectrophotometry after column chromatography with a commercial kit (code 11017, BioSystems S.A., Barcelona, Spain) following manufacturer’s instructions. The results were normalized to urinary creatinine.

### Statistical analysis

Continuous variables were summarised with means and standard errors, while qualitative variables were expressed as proportions. A logistic regression model, adjusted for sex and age, was used to test the association between *CYP* genotype and MAIP frequency. To test the association between *CYP* genotype and urinary ALA and PBG levels, a multiple lineal regression analysis was used. This regression model was adjusted for sex, age and history of acute attacks (MAIP) to avoid confusion in the results obtained, since ALA and PBG may remain elevated for many years after an acute attack. A simple test to compare proportions was made to analyse differences in allelic frequencies between MAIP and LAIP. Data were analysed using R software package (3.4.1. version).

## Results

Fifty AIP carriers aged between 16 and 77 years (44 years of mean age), 56% women, were analysed (Table 1). 78% carried the known founder pathogenic variant c.669_698del30 in *HMBS* gene. General penetrance was 52% (frequency of MAIP). Penetrance associated with the founder pathogenic variant was 48.7% (19/39 patients), which is similar to that associated with the pathogenic variant c.76C > T (50%), the second most frequent pathogenic variant in the studied cohort. Furthermore, based on population the census in our province (1,470,273 inhabitants) and the frequency of MAIP described in this study, the estimated symptomatic AIP prevalence was at least 17.7 cases/million inhabitants.

**Table 1:** Characteristics of AIP carriers

	Total (*n* = 50)	LAIP (*n* = 24; 48%)	MAIP (*n* = 26; 52%)
Age, years	44 ± 15.7	42 ± 15.8	47 ± 14.9
Women	28 (56%)	10 (42%)	18 (69%)
*HMBS* pathogenic variant			
c.669_698del30, p.(Glu223_Leu232)	39 (78%)	20 (83%)	19 (73%)
c.76C > T, p.Arg26Cys	8 (16%)	4 (17%)	4 (15%)
c.275 T > C, p.(Leu92Pro)	1 (2%)	–	1 (4%)
c.750delA, p.(Glu250GlufsTer4)	2 (4%)	–	2 (8%)

*LAIP*: Latent AIP; *MAIP*: Manifest AIP. Age shown as mean and standard deviation; sex and *HMBS* variants distribution shown as number of individuals and percentage. *HMBS* variants are referenced to NC_000011.9 (NM_000190.3)

Allele frequencies are shown in Table 2 and genotype distributions are summarized in Table 3. All genotype frequencies met the Hardy-Weinberg equilibrium. The frequency of defective *CYP2D6* alleles was 3.5 times higher in LAIP than in MAIP. MAIP was less frequent in defective allele carriers of *CYP2D6* gene (CYP2D6*4 or *5 alleles), so the possibility that a patient had MAIP was reduced by 80% on average for each additional defective allele (*4 or *5) in *CYP2D6* gene (OR 0.2; CI 95% 0.04–0.81; *p* value 0.037).

**Table 2:** *CYP* allelic frequencies

Gene	Allele	Total AIP	LAIP	MAIP	*P* value
*CYP2C9*	*2	0.28	0.33	0.23	0.255
	*3	0.01	0.02	0	
*CYP2C19*	*2	0.15	0.13	0.17	0.695
*CYP2D6*	*4	0.12	0.19	0.06	0.052
	*5	0.01	0.02	0	
*CYP3A4*	*1B	0.03	0.02	0.04	1.000
CYP3A5	*3	0.95	0.96	0.94	0.913

*MAIP* manifest AIP, *LAIP* latent AIP

**Table 3:** *Cytochrome P450* genotype distributions and MAIP frequency by genotype

Gene	Genotype	Genotype distribution		MAIP frequency			
		N	%	%	OR	CI 95%	*P* value
*CYP2C9*	two normal alleles	27	54	55.6	0.45	0.17–1.09	0.092
	one allele *2 or *3	17	34	58.8			
	two alleles *2 or *3	6	12	16.7			
*CYP2C19*	two normal alleles	35	70	48.6	1.75	0.49–6.81	0.398
	one allele *2	15	30	60.0			
	two alleles *2	0	0	–			
*CYP2D6*	two normal alleles	38	76	60.5	0.2	0.04–0.81	0.037
	one allele *4 or *5	11	22	27.3			
	two alleles *4 or *5	1	2	0			
*CYP3A4*	two normal alleles	47	94	51.1	1.94	0.14–49.63	0.629
	one allele *1B	3	6	66.7			
	two alleles *1B	0	0	–			
*CYP3A5*	two normal alleles	0	0	–	0.67	0.07–5.02	0.697
	one allele *3	5	10	60.0			
	two alleles *3	45	90	51.1			

*OR* Odds ratio adjusted for age and sex*, CI 95%* 95% confidence interval for OR, *MAIP* manifest AIP

There was also slight evidence that MAIP was reduced by each additional defective allele (*2 or 3*) in *CYP2C9* gene, although not to a statistically significant extent. When a combined *CYP2C9* and *CYP2D6* genotype was considered (data not shown), only 16.7% of individuals carrying at least one defective allele in both genes had MAIP compared with 52.2% of carriers of defective alleles in either one of the genes and with 61.9% of normal allele carriers. Thus, MAIP frequency was reduced by 65% in individuals carrying at least one defective allele in both genes (OR 0.35; CI 95% 0.12–0.89; p value 0.0368). Other different *CYP* combined genotypes were not considered since there was no evidence of individual effect in acute attacks.

The urine PBG-to-creatinine ratio (Table 4) tended to be lower in individuals carrying CYP2C9*2 or *3 and CYP2D6*4 or *5 alleles compared with respective normal allele carriers. However, this reduction was not attributed to the *CYP* genotype by itself but was associated with the history of acute attacks (*p* < 0.01). There were no differences in ALA excretion according to *CYP* genotype.

**Table 4:** Urinary ALA and PBG excretion according to genotype

Gene	Genotype	U-PBG/Cr			U-ALA/Cr		
		mean (SE)	beta (CI 95%)	*P* value	mean (SE)	beta (CI 95%)	*P* value
*CYP2C9*	two normal alleles	12.93 (3.36)	−3.82 (−9.25;-1.62)	0.163	8.48 (1.30)	−1.26 (−8.07;5.55)	0.711
	one allele *2 or *3	5.62 (1.88)			12.85 (6.67)		
	two alleles *2 or *3	1.87 (0.80)			4.98 (0.71)		
*CYP2C19*	two normal alleles	9.12 (2.34)	−1.84 (− 10.11; 6.43)	0.655	10.58 (3.01)	−3.96 (− 14.03;6.11)	0.432
	one allele *2	9.32 (4.27)			6.39 (1.30)		
	two alleles *2	NA			NA		
*CYP2D6*	two normal alleles	10.71 (2.63)	−0.67 (−8.60; 7.27)	0.866	10.45 (2.94)	−3.69 (−13.34;5.96)	0.444
	one allele *4 or *5	5.34 (2.38)			5.93 (1.05)		
	two alleles *4 or *5	0.76 (NA)			11.82 (NA)		
*CYP3A4*	two normal alleles	9.52 (2.12)	−7.11 (−25.30;11.09)	0.435	9.67 (2.27)	−6.72 (−29.08;15.65)	0.547
	one allele *1B	1.88 (1.07)			2.96 (1.56)		
	two alleles *1B	NA			NA		
*CYP3A5*	two normal alleles	NA	2.54 (−10.64;15.72)	0.699	NA	5.46 (−10.63;21.54)	0.497
	one allele *3	6.45 (5.00)			4.33 (1.02)		
	two alleles *3	9.45 (2.2)			9.86 (2.38)		

*U-PBG/Cr* Urine PBG-to-creatinine ratio (μmol /mmol), reference range < 1.5 μmol /mmol, *U-ALA/Cr* Urine ALA-to-creatinine ratio (μmol /mmol), reference range < 3.8 μmol /mmol, *SE* Standard error; beta: regression analysis coefficient adjusted by age, sex and history of acute attacks; CI 95%: 95% confidence interval of beta; *NA*: Not applicable

## Discussion

Several clinical studies have described a penetrance of around 10–20% in AIP [2]. Other studies based in the prevalence of pathogenic variants in *HMBS* gene in the general populations have estimated a penetrance of below 1% [5, 6]. However, there are high-penetrant mutations such as R173W (50%) and W198X (44%) [7], which are highly frequent in Nova Scotia (Canada) [25] and Sweden [26], respectively, due to a founder effect. Here, we describe the high clinical penetrance associated with the founder mutation c.669_698del30 in *HMBS* gene [27], which is frequent in southern Spain. This penetrance is similar to that of the W198X mutation, which is associated with a high prevalence of AIP in Sweden (23 cases/million inhabitants). In addition, we estimated the prevalence of AIP in the Region of Murcia to be about 3 times higher than that estimated in Spain as a whole (6.3 cases/million inhabitants) [1]. All the above suggest that, while low-penetrant *HMBS* mutations may be frequent in a healthy population, frequent high-penetrant mutation might be responsible for MAIP in populations with a high prevalence of AIP. High-penetrant AIP mutations may determine the genetic counselling offered to a specific population and carriers should thereby benefit from specific health care planning.

It is also of great interest to recognize how the genetic background may modulate the penetrance, making personalized genetic counselling and health care more feasible. To date, a high prevalence of specific *CYP* alleles in some types of porphyria has been described compared to that observed in a healthy population, suggesting that they might be susceptibility factors [20–22]. The present study provides further evidence that *CYP* genes may constitute penetrance-modifying factors in AIP, since CYP2D6*4 and *5 were more frequent in LAIP than in MAIP, although their allelic frequencies were similar in whole AIP carriers and the general population [27]. The differential distribution of CYP2D6*4 and *5 alleles between MAIP and LAIP supports the hypothesis that *CYP2D6* may be associated with acute clinical manifestation of AIP. Accordingly, MAIP was less frequent in defective CYP2D6*4 and *5 allele carriers.

Furthermore, for the first time, the frequency of CYP2C9*2, *3; CYP2C19*2; CYP3A4*1B and CYP3A5*3 alleles has been determined in an AIP population, in which it was found to be similar to that of the general population and between MAIP and LAIP [27–29]. However, in our opinion, we cannot dismiss a possible relationship of these genes with AIP, which would require larger studies.

Our results suggest that CYP2D6*4 and *5 defective alleles play a protective role in the clinical onset of AIP, modulating its penetrance. AIP patients with defective *CYP2D6* alleles may be less susceptible to porphyrogenic xenobiotic intermediate metabolites. Another possible explanation is that defective *CYP2D6* alleles might consume less haem than normal alleles, so defective *CYP* allele carriers might be less prone to the overstimulation of haem synthesis. This last hypothesis would be supported if baseline levels of ALA and PBG were lower in defective *CYP2D6* allele carriers. However, we were unable to demonstrate that lower urinary ALA and PBG levels in defective *CYP2D6* allele carriers are directly associated with *CYP* genotype rather than acute attack history.

The strength of this study lies in the high genetic homogeneity in terms of a causal AIP mutation in *HMBS* gene, which eliminates biases due to differences in penetrance associated with various mutations. However, this study is not without limitations. The low allele frequencies of the *CYP* genetics variants and the limited number of patients studied make it necessary to confirm these findings in larger AIP populations. Further studies which include other *CYP* genes, as well as other candidate genes using next generation sequencing, might provide a more comprehensive overview of their possible modulating role in AIP.

## Conclusions

AIP prevalence in the Spanish Region of Murcia is 17.7 cases/million inhabitants, almost 3 times higher than that estimated for the rest of Spain. The penetrance is 52%, similar to other founder mutation AIP populations. CYP2D6*4 and *5 alleles may be protective factors for acute attacks, and *CYP2D*6 may constitute a penetrance-modifying gene. Further studies are needed to confirm these findings, which would allow a further progress in AIP clinical risk profile assessment.

We highlight the relevance of knowing the prevalence and penetrance of AIP in a given population, especially in that ones with high prevalence of specific *HMBS* mutations, since published data vary among populations and mutations. These results will have a positive impact on AIP carriers genetic counselling and will allow a better planning of health care resources in our population. Furthermore, we emphasize the potential of applying *CYP* genotyping in AIP precision medicine, enabling personalized risk stratification of acute clinical manifestation based on individual *CYP* allelic profile and leading to predictive personalized medicine for each AIP carrier in the future.

## Abbreviations

AIP: Acute intermittent porphyria
ALA: Aminolevulinic acid
ALAS1: Aminolevulinic acid synthase 1
CYP: Cytochrome P450
HMBS: Hydroxymethylbilane synthase
LAIP: Latent acute intermittent porphyria
MAIP: Manifest acute intermittent porphyria
PBG: Porphobilinogen
